# Single-Cavity Dual-Comb Mueller Polarimetry for Material-Aware LiDAR

**DOI:** 10.1021/acsomega.6c08407

**Published:** 2026-10-01

**Authors:** Hani J. Kbashi, Sergey Sergeyev

**Affiliations:** † 1722 Aston University, Aston Institute of Photonic Technologies, Birmingham B4 7ET, U.K.; ‡ University of Warith Al-Anbiyaa, Department of Medical Physics, College of Science, Karbala Road, Karbala 5600, Iraq

## Abstract

We demonstrate a single-cavity dual-comb Mueller-matrix polarimetric LiDAR that rapidly retrieves full 4 × 4 Mueller matrices without external polarization-state generators or polarization-state analyzers. The key advance is that the intrinsic polarization beating of the polarization-multiplexed dual-combs from the single source provides a self-scanning incident Stokes-state basis, replacing the sequential polarization-state generation normally required in active Mueller polarimetry. The recovered Mueller matrices are decomposed into physically interpretable descriptors, including diattenuation, polarizance, retardance, depolarization, and polarimetric-purity indices, to construct a multidimensional material-fingerprint space. Experiments on aluminum, wood, plastic, and mirror targets show clear separation of material classes in this physics-based feature space. Combining convex-hull analysis with machine-learning classification yields 93.75% accuracy. These results establish dual-comb Mueller-matrix polarimetry as a compact and practical framework for material-aware remote sensing and other applications requiring interpretable polarization contrast.

## Graphical abstract

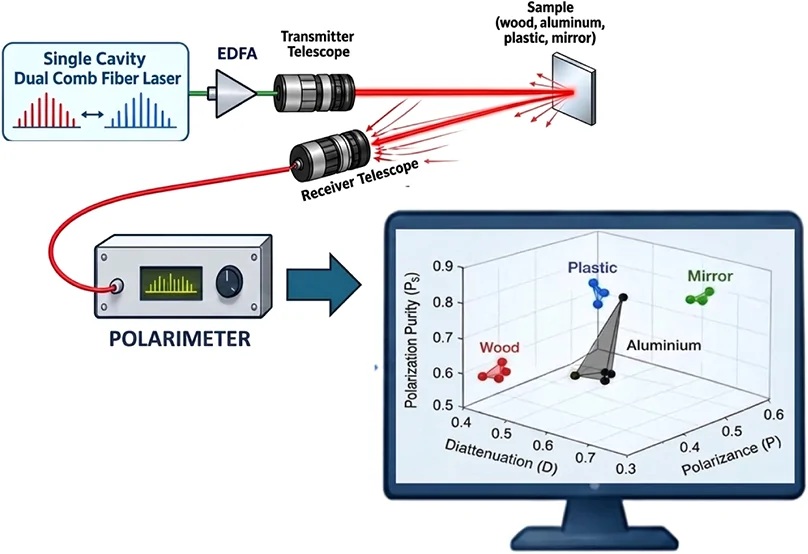

## Introduction

Polarimetric light detection and ranging (LiDAR) extends conventional ranging by probing how optical fields interact with the microstructure, anisotropy, and scattering properties of a target. Whereas intensity-only LiDAR primarily reports distance and reflectance, polarization-resolved measurements can reveal information about surface texture, preferred orientation, and depolarizing behavior that is inaccessible to scalar sensing.^1–6^ The natural framework for describing these interactions is provided by the Stokes vector and Mueller matrix, which together capture the complete transformation imposed on an incident polarization state by a material or interface.^7–13^ This added sensitivity has motivated growing interest in polarimetric sensing across materials characterization,^14–16^ environmental monitoring,^1–6^ biomedical optics,^17–20^ and structural diagnostics.^21–24^ Yet robust material identification in LiDAR remains difficult because targets with similar reflectance can exhibit markedly different polarization signatures, while measurement geometry, roughness, and noise can obscure simple intensity-based contrasts.

Although Mueller-matrix polarimetry is well established in laboratory imaging and optical characterization, its use in active LiDAR is still limited. Many existing systems measure only part of the polarization response, require several sequential measurements, or depend on external polarization-state generators and analyzers. These requirements make the instruments more complex, slow down data acquisition, and make practical use outside the laboratory more difficult.^1^ The challenge is especially important when the full Mueller matrix must be measured quickly, for example in dynamic scenes or field-deployable sensing. Unlike conventional dual-comb ranging systems that usually rely on two synchronized comb lasers,^24,25^ single-cavity dual-comb photonics offers a more compact route. In this approach, two mutually coherent combs are generated in the same laser cavity and can provide high timing precision together with built-in polarization diversity.^26–33^ In particular, the beating between two orthogonally polarized combs naturally produces a sequence of different polarization states, removing the need for external polarization modulation.^28^ This creates an opportunity for fast and stable Mueller-matrix acquisition with simpler hardware. However, a LiDAR system in which a single-cavity dual-comb source itself provides the required polarization diversity for full Mueller-matrix recovery has not previously been established for material-resolved remote sensing. Here, we introduce such self-scanning Mueller-matrix LiDAR based on a single-cavity polarization-multiplexed dual-comb fiber laser. Instead of generating incident and analyzing polarization states one by one with external optics, the system uses the intrinsic polarization dynamics of the dual-comb source as the measurement basis. This enables full Mueller-matrix reconstruction with compact hardware and without external polarization switching. We then convert the recovered matrices into physically meaningful descriptors, including diattenuation, polarizance, retardance, depolarization, and polarimetric purity. These descriptors form a multidimensional material-fingerprint space. Using aluminum, wood, plastic, and mirror targets, we show that different materials form clearly separated clusters and that lightweight classification reaches 93.75% accuracy. The main contribution of this work is therefore not only material classification, but a compact dual-comb photonic route to full-vector, interpretable, material-aware LiDAR. The work advances the field in three connected ways: first, it uses a single-cavity dual-comb laser as an intrinsic source of polarization states; second, it converts dual-comb temporal beating into a basis for full Mueller-matrix acquisition; and third, it maps the recovered matrices onto interpretable descriptors of light–matter interaction for material-aware LiDAR.

## Experimental Set-Up and Validation

Figure 1 shows the experimental platform for dual-comb polarimetric LiDAR. The present experiment uses a fixed, noncoaxial transmitter–receiver geometry with stationary targets. The transmitter and receiver optical axes were manually aligned so that their fields of view overlapped at the selected target positions. Beam-steering-dependent receiver alignment was therefore not addressed in this proof-of-concept study.

**Figure 1:**
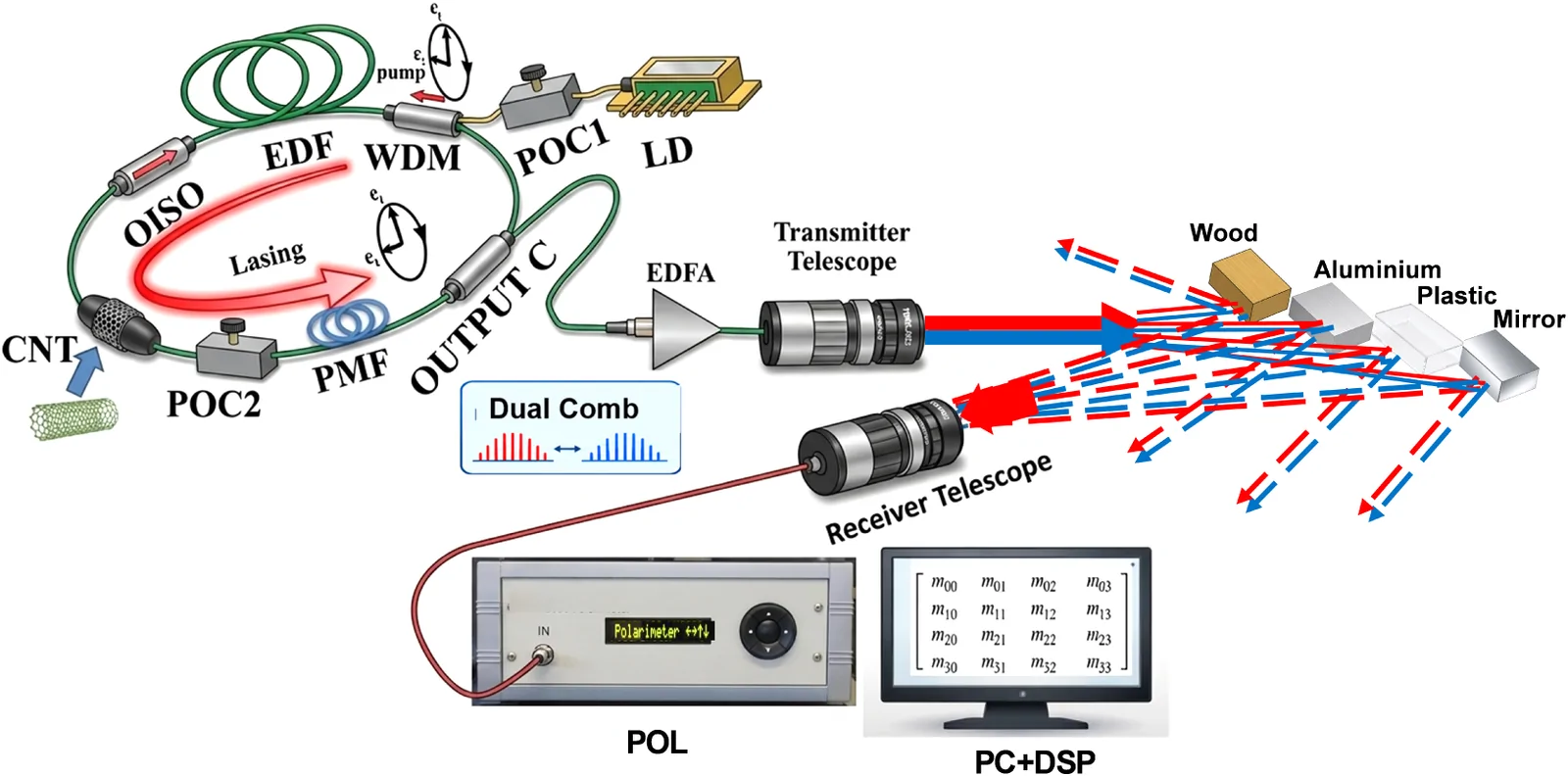
Dual-comb fiber laser LiDAR sensing: WDM: wavelength division multiplexing, LD: pump laser diode, EDF: erbium doped fiber, OISO: optical isolator CNT: carbon nanotubes-based saturable absorber, POC1 and POC2: polarization controllers, PMF: polarization maintaining fiber, EDFA: Erbium doped fiber amplifier, POL: Fast Polarimeter, PC+DSP: Personal computer with digital signal processing software.

The source is a single-cavity erbium-fiber laser mode locked with a carbon-nanotube saturable absorber and pumped at 980 nm through wavelength-division multiplexing. A 30 cm polarization-maintaining fiber segment introduces birefringence that supports two mutually coherent pulse trains with slightly different repetition rates and orthogonal polarization states. Unidirectional operation is enforced by a 51 dB polarization-independent isolator, while intracavity polarization controllers tune the operating point. A 90:10 coupler closes the cavity and extracts the measurement output signal. The total cavity length is 5.66 m and the output signal power is 3 mW. In the dual-comb regime, the slow- and fast-axis pulse trains operated at repetition rates of 35.286201 and 35.285016 MHz, respectively, giving a repetition-rate offset of 1.185 kHz and a polarization-refresh period of 0.84388 ms. The corresponding RF signal-to-noise ratios were approximately 87 and 82 dB for the two combs, confirming stable dual-comb operation during polarimetric acquisition.

At the output, the two combs overlap in generating a radiofrequency heterodyne beating signal with intrinsic polarization modulation (see details in Supporting Information). The signal is amplified to 33 dBm with an erbium-doped fiber amplifier and directed to the target. Backscattered light is collected with a fiber collimator and analyzed by a fast polarimeter (PM1000, Novoptel), which records the full Stokes vector in real time. By sampling the polarization evolution over successive beat periods, we construct the reference and scattered Stokes matrices and retrieve the full Mueller matrix of the target (see details in Supporting Information). This architecture removes the need for external polarization-state generators and analyzers, reducing system complexity while improving acquisition speed and stability. This operating principle differs fundamentally from sequential Mueller polarimetry. Rather than preparing a finite set of polarization states with external modulators, the dual-comb source continuously traces a reproducible trajectory on the Poincaré sphere through the heterodyne beating of two mutually coherent, orthogonally polarized combs. Selected points along this trajectory provide the linearly independent incident Stokes states required for Mueller-matrix inversion. The single-cavity configuration is important because both combs share the same environmental perturbations, improving mutual coherence and reducing differential drift between the polarization-state basis and the measurement channel.

### Dual-Comb Polarization Dynamics and Stokes-State Sampling

Figure 2 shows the time-domain Stokes dynamics generated by heterodyne beating of the slow and fast dual-comb pulse trains. The fast polarimeter records the full Stokes vector (S_0_–S_3_) in real time, revealing equally spaced polarization-modulated bursts with a temporal separation of 0.843 ms, corresponding to a repetition-rate offset of 1.185 kHz. Measurements were acquired in single-shot mode over an 80 ms window with 80 ns temporal resolution (12.5 MHz sampling), providing approximately 95 refresh cycles per trace. This periodic modulation supplies a reproducible sequence of polarization states without external polarization switching.

**Figure 2:**
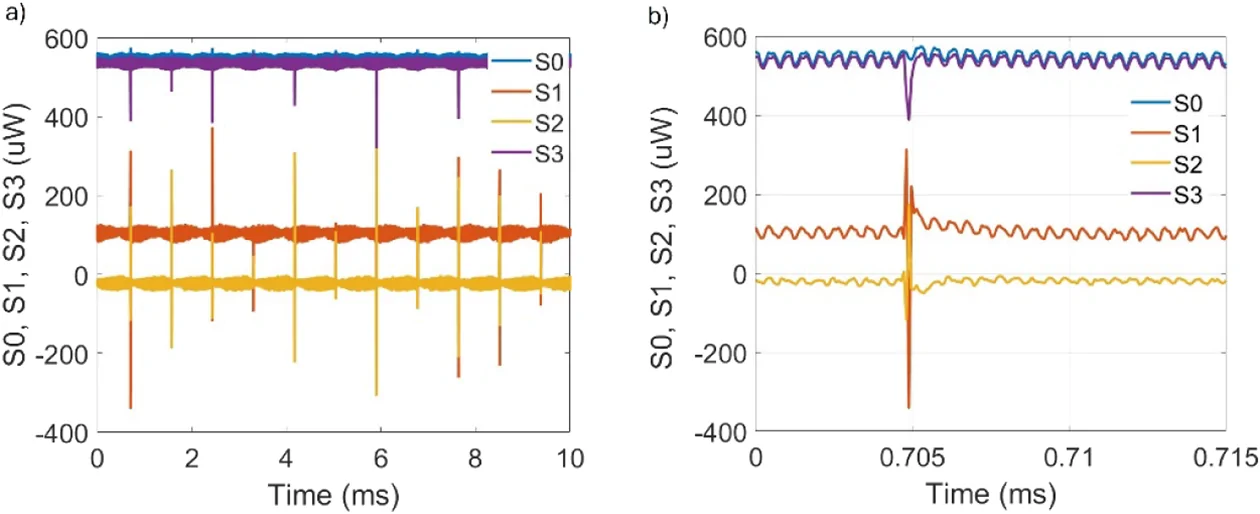
Time-domain dynamics of the Stokes parameters (S_0_–S_3_) of the dual-comb signal measured with the fast polarimeter, illustrating polarization-resolved heterodyne beating. (a) Beating sequence over a 10 ms time span. (b) Internal structure of a single burst on the corresponding time scale.

Four representative Stokes states were selected from each beat cycle to assemble the reference matrix, and the corresponding scattered states were measured from the target return to reconstruct the full Mueller matrix (Figure 2 a and b). The selected states were chosen to maximize polarization diversity and to form a well-conditioned reference Stokes matrix for stable Mueller-matrix inversion. In this sense, the dual-comb waveform provides a self-generated polarization sampling basis, converting temporal heterodyne dynamics into vectorial polarimetric information. This sampling strategy exploits the intrinsic polarization evolution of the dual-comb source, avoiding external state generators or analyzers while maintaining high repeatability. We applied the method to aluminum, wood, plastic, and mirror targets, with five repeated measurements per material. The resulting Mueller matrices form the basis for the purity metrics, Lu–Chipman decomposition, and classification analyses presented below.

### Self-Scanning Mueller Acquisition Using Dual-Comb Polarization Beating

The present architecture converts the intrinsic polarization beating of the dual-comb source into a self-scanning Mueller acquisition process. In conventional active Mueller polarimetry, at least four independent incident states and four analyzer states are usually generated by external polarization optics or electro-optic modulation. Here, the polarization-multiplexed dual-comb source supplies the incident-state diversity directly, while the fast Stokes polarimeter records the complete returned polarization vector. This removes the need for synchronized polarization switching and reduces the measurement to the inversion of reference and scattered Stokes matrices sampled from the same reproducible dual-comb cycle. The approach therefore combines coherent dual-comb timing, polarization multiplexing, and full-vector polarimetric recovery in a single compact LiDAR platform.

### Extraction of Polarimetric Purity Indices

The polarization state of light is described using the Stokes vector

1 $$S=\left[\begin{array}{l} S_{0}\\ S_{1}\\ S_{2}\\ S_{3} \end{array}\right]$$

where *S*_0_ represents total power, *S*_1_ describes linear polarization in the horizontal and vertical directions, *S*_2_ represents linear polarization at ±45°, and *S*_3_ describes circular polarization. The scattered Stokes vectors from each material are measured and used to calculate the Mueller matrix, which describes the polarization transformation caused by the material. The relationship between incident (reference *S_R_*) and scattered *S_SC_* polarization states is expressed as

2 $$S_{SC}=MS_{R}$$

3 $$S_{SC}=\left[\begin{array}{llll} S_{SC00} & S_{SC01} & S_{SC02} & S_{SC03}\\ S_{SC10} & S_{SC11} & S_{SC12} & S_{SC13}\\ S_{SC20} & S_{SC21} & S_{SC22} & S_{SC23}\\ S_{SC30} & S_{SC31} & S_{SC32} & S_{SC33} \end{array}\right],M=[\begin{array}{llll} m_{00} & m_{01} & m_{02} & m_{03}\\ m_{10} & m_{11} & m_{12} & m_{13}\\ m_{20} & m_{21} & m_{22} & m_{23}\\ m_{30} & m_{31} & m_{32} & m_{33} \end{array}],S_{R}=[\begin{array}{llll} S_{R00} & S_{R01} & S_{R02} & S_{R03}\\ S_{R10} & S_{R11} & S_{R12} & S_{R13}\\ S_{R20} & S_{R21} & S_{R22} & S_{R23}\\ S_{R30} & S_{R31} & S_{R32} & S_{R33} \end{array}]$$

where ***M*** is the Mueller matrix that contains the complete polarization information about the target materials and enables extraction of physical optical properties.

As follows from,^7,12,24^ Mueller matrix ***M*** in (eq 3) can be written in the following partitioned

form:

4 $$\mathrm{M̂}=\frac{M}{m_{00}}=m_{00}\left(\begin{array}{ll} 1 & D^{T}\\ P & m \end{array}\right)$$

Where

5 $$M=S_{\mathrm{SC}}S_{R}^{-1},P\equiv \frac{1}{m_{00}}{\left(m_{10},m_{20},m_{30}\right)}^{T},D\equiv \frac{1}{m_{00}}{\left(m_{01},m_{02},m_{03}\right)}^{T},m\equiv \frac{1}{m_{00}}\left(\begin{array}{lll} m_{11} & m_{12} & m_{13}\\ m_{21} & m_{22} & m_{23}\\ m_{31} & m_{32} & m_{33} \end{array}\right)$$

Here ***D*** and ***P*** are the diattenuation and polarizance vectors where the lengths of these vectors *D* = | ***D*** | *P* = | ***P*** | are diattenuation and polarizance. The mean transmittance for incident unpolarized light for matrix ***M*** is *m*_00_ and the degree of polarimetric purity is characterized by the depolarization index *P_d_*:^7,12,24^

6 $$P_{d}=\sqrt{D^{2}+P^{2}+{P_{s}}^{2}}$$

Here *P_s_* = |*m*_2_| is Euclidean norm of the submatrix ***m***, called polarization structure.^7,12,24^

By using eqs 3–6, the *D*, *P* and *P_s_* can be found as follows:^7,12,24^

$$D=\sqrt{\frac{\overset{3}{\underset{j=1}{\sum }}m_{0j}^{2}}{m_{00}^{2}}},P=\sqrt{\frac{\overset{3}{\underset{i=1}{\sum }}m_{i0}^{2}}{m_{00}^{2}}},P_{s}=|m_{2}|=\sqrt{\frac{\overset{3}{\underset{i,j=1}{\sum }}m_{ij}^{2}}{3m_{00}^{2}}}$$ 7

If Mueller matrix is nondepolarizing, *P_d_* = 1 and for nonpure or depolarizing Mueller matrix, *P_d_* < 1.^7,12,24^

### Lu-Chipman Decomposition

These parameters provide physically meaningful optical properties with initial polarization-based material characterization. The Mueller matrices are further decomposed using the Lu-Chipman decomposition, which separates the Mueller matrix into three physically interpretable matrices corresponding to depolarizer (*M*_Δ_), a retarder (*M_R_*), and a diattenuator (*M_D_*):^8,11,18^

8 $$M=M_{\Delta }M_{R}M_{D}$$

This decomposition provides intrinsic optical properties that are independent of measurement configuration and directly related to material structure. The corresponding matrices take the following form^8,11,18^

9 $$M_{\Delta }=\left[\begin{array}{ll} 1 & {0⃗}^{T}\\ {\mathrm{P⃗}}_{\Delta } & m_{\Delta } \end{array}\right],M_{R}=\left[\begin{array}{ll} 1 & {0⃗}^{T}\\ 0⃗ & m_{R} \end{array}\right],M_{D}=\left[\begin{array}{ll} 1 & {0⃗}^{T}\\ \mathrm{D⃗} & m_{D} \end{array}\right]$$

The diattenuation magnitude and diattenuation vector can be found from eqs 5 and 7 and 3 × 3 submatrix *m_D_* can be found as follows:

10 $$m_{D}=\sqrt{1-D^{2}}\cdot I+\frac{1-\sqrt{1-D^{2}}}{D^{2}}\cdot \mathrm{D⃗}{\mathrm{D⃗}}^{T}$$

Here ***I*** is the identity matrix.

Next, the product of ***M***_Δ_ and ***M**_R_* is calculated:

11 $$M_{\Delta }\cdot M_{R}=M\cdot M_{D}^{-1}=M^{\prime }$$

As follows from eq 11, if *m* is 3 × 3 submatrix of ***M*** and *m*′ is the submatrix of *m*′, then

12 $$m^{\prime }=m_{\Delta }m_{R}\;\mathrm{and}\;m_{\Delta }^{2}=m^{\prime }\cdot {\left(m^{\prime }\right)}^{T}$$

By using Cayley-Hamilton theorem, the submatrix ***m***_Δ_ can be found as follows:^18^

13 $$m_{\Delta }=\pm {\{m^{\prime }\cdot {\left(m^{\prime }\right)}^{T}+\left(\sqrt{\lambda_{1}\lambda_{2}}+\sqrt{\lambda_{3}\lambda_{2}}+\sqrt{\lambda_{1}\lambda_{3}}\right)\cdot I\}}^{-1}\cdot \left\{\left(\sqrt{\lambda_{1}}+\sqrt{\lambda_{2}}+\sqrt{\lambda_{3}}\right)\cdot m^{\prime }\cdot {\left(m^{\prime }\right)}^{T}+\sqrt{\lambda_{1}\lambda_{2}\lambda_{3}}\cdot I\right\}$$

Here *λ*_1_, *λ*_2_, *λ*_3_ are eigenvalues of ***m*** ′·(***m*** ′)^*T*^ matrix.

Also, $\overset{\to }{P_{\Delta }\;}$ can be found in the form:^18^

14 $$\overset{\to }{P_{\Delta }\;}=\frac{\mathrm{P⃗}-m\cdot \mathrm{D⃗}}{1-D^{2}}$$

where polarizance vector *P⃗* can be found from eq 5.

After computing all elements of matrix *M*_Δ_, the net depolarisation Δ can be found as

15 $$\Delta =1-\left(\frac{\left|trM_{\Delta }-1\right|}{3}\right)$$

Finally, the retardance matrix *M_R_* and retardance *R* take the form:

16 $$M_{R}=M_{\Delta }^{-1}\left(M^{’}\right),R=\cos^{-1}\left(\frac{trM_{R}}{2}-1\right)$$

## Results and Discussion

Having established that the dual-comb waveform provides the polarization-state basis for full Mueller-matrix recovery, we next examine whether the retrieved matrices contain material-specific physical information. The purpose of the following analysis is therefore not simply to classify four samples, but to test whether self-scanned dual-comb Mueller polarimetry can expose interpretable light–matter-interaction signatures. Because the present data set contains four representative materials measured under controlled conditions, the classification analysis should be interpreted as proof-of-concept validation of the self-scanned Mueller feature space rather than as a large-scale machine-learning benchmark.

### Polarimetric Purity Indices as First Level Material Fingerprints

As a first discrimination layer, we extracted the polarimetric purity metrics diattenuation (D), polarizance (P), polarization structure (P_s_), and depolarization (P_d_) from the measured Mueller matrices. Figure 3 shows that mirror and aluminum occupy the high-D/low-depolarization regime, consistent with specular or metallic reflection that largely preserves polarization, whereas wood and plastic exhibit lower D and higher P_d_, indicating stronger multiple scattering and reduced polarization memory. Plastic shows the largest P, while mirror exhibits the highest P_s_ and P_d_, highlighting that the four materials are not distinguished by a single scalar observable but by a combined polarimetric signature. These trends establish purity indices as compact, physically interpretable fingerprints for first-pass material separation. Thus, the purity descriptors do not merely provide numerical separation; they encode material-dependent amplitude anisotropy, polarization retention, and depolarizing scattering, which are the physical mechanisms targeted by full-vector polarimetric LiDAR.

**Figure 3:**
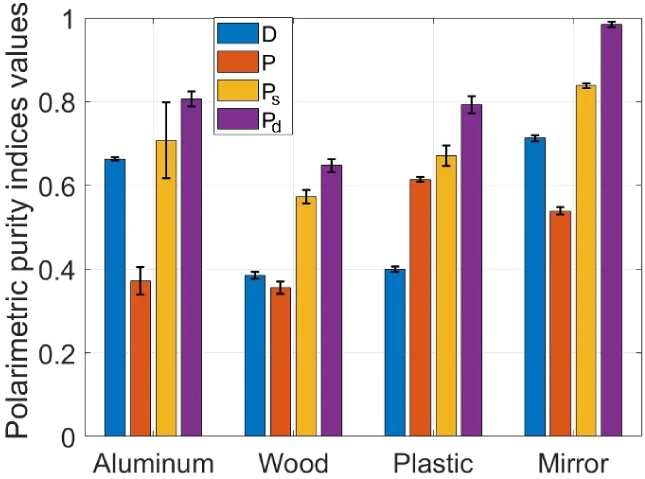
Mean values and standard deviations of polarization purity parameters including diattenuation (D), polarizance (P), global measure of polarization structure (P_s_), and global measure of depolarization (P_d_) for aluminum, wood, plastic, and mirror measured using the polarimetric dual-comb LiDAR system. Error bars represent standard deviation across repeated measurements.

To test whether these metrics provide separable fingerprints, we embedded the measurements in the three-dimensional space spanned by D, P, and P_s_ (Figure 4a). The four materials form distinct clusters, with metallic/specular samples displaced from diffuse samples primarily along the depolarization-sensitive directions. Convex-hull classification in this space (Figure 4b) shows little overlap between classes, indicating that physics-based discrimination is feasible without model training. This model-free separation is important because it shows that the dual-comb Mueller descriptors generate physically separable material clusters before any machine-learning optimization is applied. Table 1 summarizes the metric values and their physical interpretation for each material.

**Figure 4:**
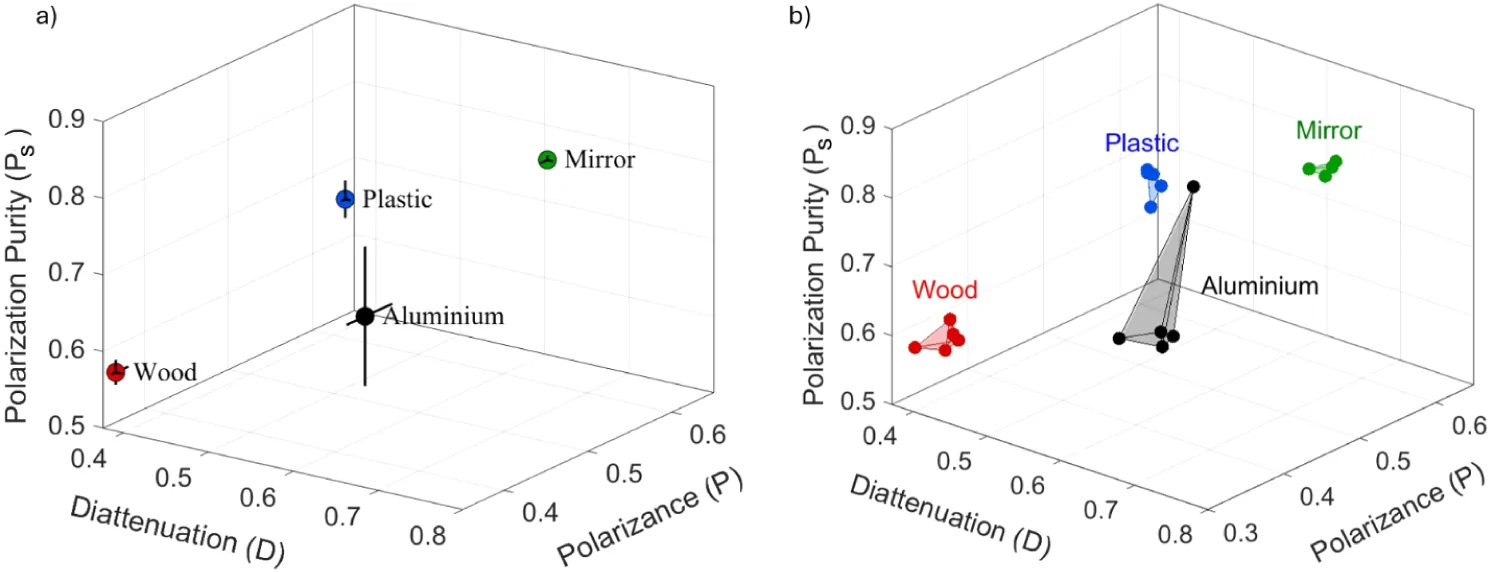
(a) Three-dimensional material-fingerprint space constructed from Mueller-matrix-derived diattenuation, polarizance, and polarization-structure parameters. Error bars indicate measurement uncertainty. (b) Convex-hull representation of the D–P–Ps feature space, illustrating the class boundaries and cluster separation for aluminum, wood, plastic, and mirror.

**Table 1:** Classification of Materials Based on Polarization Purity

Material	D	P	P_s_	P_d_	Physical explanation
Aluminum	0.6644	0.3707	0.716675	0.6078	Strong metallic reflection, small roughness, surface-dependent polarization.
Wood	0.3843	0.3559	0.57612	0.65073	Multiple scattering, fibrous structure.
Plastic	0.4009	0.61325	0.68027	0.801125	Birefringence and moderate scattering and polarization effects.
Mirror	0.7118	0.540125	0.83982	0.98562	Preserves polarization-induces phase shift (retardance), specular reflection, minimal scattering, small depolarization.
Highest to lowest	Mirror, Aluminum, Plastic, Wood	Plastic, Mirror, Aluminum, Wood	Mirror, Aluminum, Plastic, Wood	Mirror, Aluminum, Plastic, Wood	

### Principal Component Analysis of Polarization Fingerprints

To evaluate the dimensionality and separability of the polarization fingerprints, we applied principal component analysis (PCA) to the purity-derived feature set. The projection onto the first two principal components (Figure 5a) produces well-separated clusters for aluminum, wood, plastic, and mirror, showing that most of the material-specific variance is retained in a low-dimensional representation. The corresponding variance spectrum (Figure 5b) shows that PC1, PC2, and PC3 account for 71.26%, 25.84%, and 2.90% of the variance, respectively, so the first two components capture 97.10% of the total variance. The high variance captured by the first two components indicates that the dominant material contrast is concentrated in a compact combination of Mueller-derived physical parameters rather than distributed across many empirical observables. This strong compression makes PCA a suitable preprocessing step for classification while preserving the structure of the physics-based fingerprint space.

**Figure 5:**
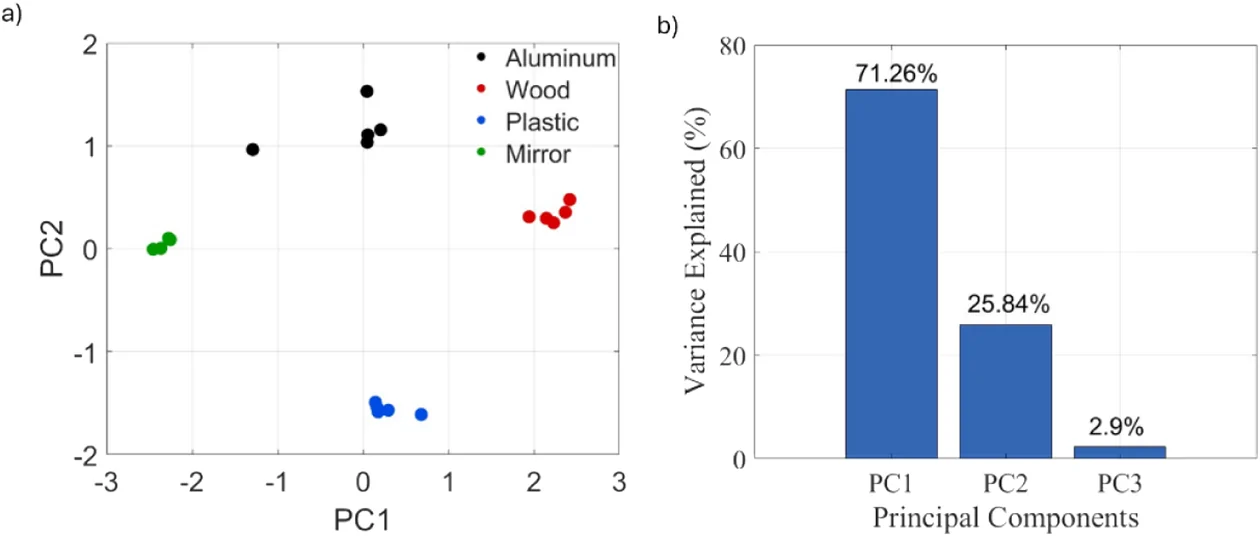
(a) Principal component analysis projection of material fingerprints showing separation between aluminum, wood, plastic, and mirror, (b) PCA variance contribution values.

### Machine Learning Classification Performance

The machine-learning analysis serves as a secondary validation step, not as the primary source of material contrast. The contrast originates physically from the full 4 × 4 Mueller matrix retrieved by the dual-comb polarimetric architecture; the classifier tests whether these physics-based features provide a separable representation of material classes in a compact, automated way. The analysis pipeline is (1) Mueller-matrix reconstruction from the reference and scattered Stokes matrices; (2) extraction of diattenuation (D), polarizance (P), polarization structure (P_s_), and depolarization index (P_d_); (3) principal component analysis (PCA) to reduce feature redundancy while preserving intermaterial variance; and (4) k-nearest-neighbors (k-NN) classification on the PCA-reduced features. k-NN was selected because it is nonparametric, makes no distributional assumptions about the feature clusters, and provides decision boundaries consistent with the convex-hull geometry of the physics-based feature space (see Supporting Information, Section S2, for the details).

### PCA Variance Decomposition

The projection onto the first two principal components captures 97.10% of the total intermaterial variance: PC1 accounts for 71.26% (dominant axis: specular vs diffuse contrast, driven by covariation of D and P_s_) and PC2 for a further 25.84% (secondary axis: polarizance vs depolarization contrast, driven by P and P_d_). The residual PC3 contributes only 2.90% and is negligible for classification. This strong compression into two dimensions confirms that the dominant material contrast is concentrated in a compact combination of Mueller-derived physical parameters (Figure 5b), and that the k-NN classifier operates in a well-conditioned, effectively two-dimensional feature space.

### Classifier Performance and Confusion Analysis

The confusion matrix in Figure 6 shows strong diagonal dominance and an overall proof-of-concept accuracy of 93.75%. Wood, plastic, and mirror were classified without error. The residual confusion involves aluminum, which was misclassified against mirror in some trials. This is physically interpretable: both aluminum and mirror are specular, polarization-preserving surfaces that occupy adjacent positions in the D–P–Ps feature space (D: 0.664 vs 0.712; Ps: 0.717 vs 0.840, Table 1). Their intracluster separation along PC2 (driven primarily by P: 0.371 vs 0.540, and Pd: 0.608 vs 0.986) is narrower than the within-class scatter for the small data set (N = 5 per class), so the confusion arises precisely at the physically expected boundary between the two most optically similar materials. No material pair that is well-separated in the feature space is confused by the classifier, confirming that the machine-learning output reflects the Mueller-matrix-derived feature geometry rather than a classifier artifact. Including the Lu–Chipman retardance as an additional feature is expected to improve aluminum/mirror separation in future extended data sets.

**Figure 6:**
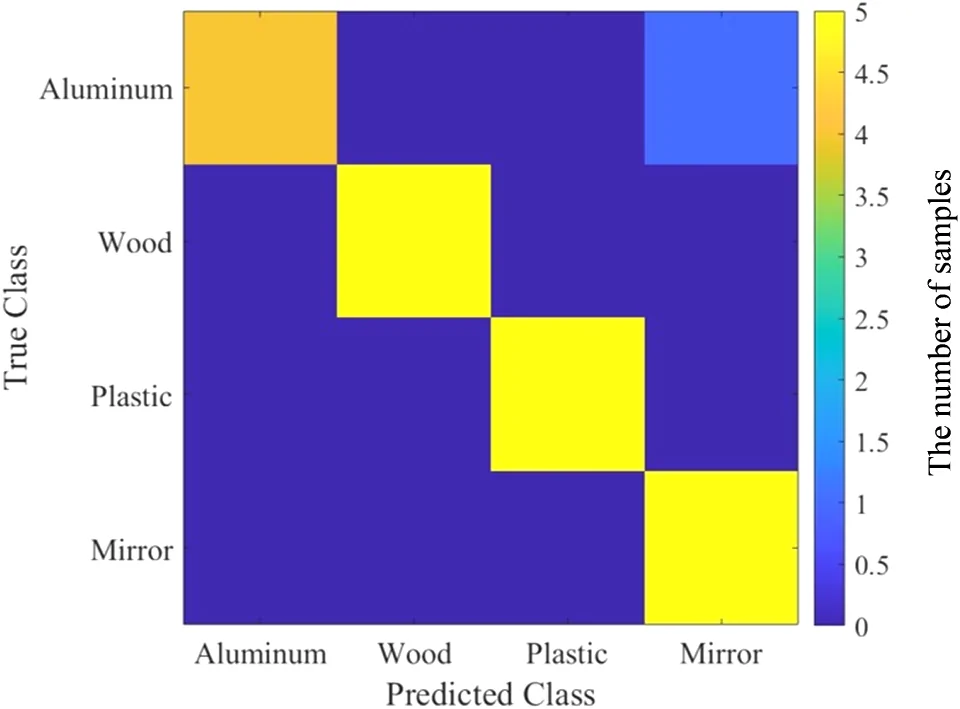
Confusion matrix showing the classification of aluminum, wood, plastic, and mirror using PCA-reduced, Mueller-matrix-derived polarimetric fingerprints with a k-nearest-neighbors (k-NN) classifier (k = 3, leave-one-out cross-validation). Diagonal entries give the number of correctly classified samples; off-diagonal entries give the number of misclassified samples. All misclassifications occur between aluminum and mirror, consistent with their proximity in the D–P–Ps feature space. Color bar: number of samples.

### Limitations of the Proof-of-Concept Data Set

The data set contains 20 measurements (5 per material, 4 materials) acquired at a single wavelength (1550 nm band), a single incidence angle, and a fixed target distance. The reported 93.75% accuracy is a proof-of-concept estimate of the discriminability of the self-scanned Mueller feature space; it should not be interpreted as a general field-performance figure. Rigorous benchmarking would require independent training and test sets, repeated k-fold cross-validation, and measurements spanning different geometries, surface finishes, environmental conditions, wavelengths, and a broader material set. These are identified as essential next steps in Discussion and Outlook.

### Intrinsic Optical Parameters from Lu–Chipman Decomposition

Lu–Chipman decomposition provides a complementary set of intrinsic descriptors—diattenuation, retardance, and depolarization—that separate the materials in a physically interpretable way (Figure 7a). Aluminum and mirror show the largest diattenuation, consistent with polarization-preserving specular reflection, whereas wood and plastic exhibit reduced diattenuation and stronger depolarizing behavior associated with diffuse or volumetric scattering. Wood shows the largest retardance, consistent with anisotropic fibrous structure, while plastic exhibits intermediate retardance, likely reflecting weaker internal anisotropy. Embedding these quantities in the three-dimensional space of diattenuation, retardance, and depolarization (Figure 7b) again yields distinct material clusters, confirming that Lu–Chipman-derived parameters provide a compact intrinsic fingerprint that complements the purity-based analysis.

**Figure 7:**
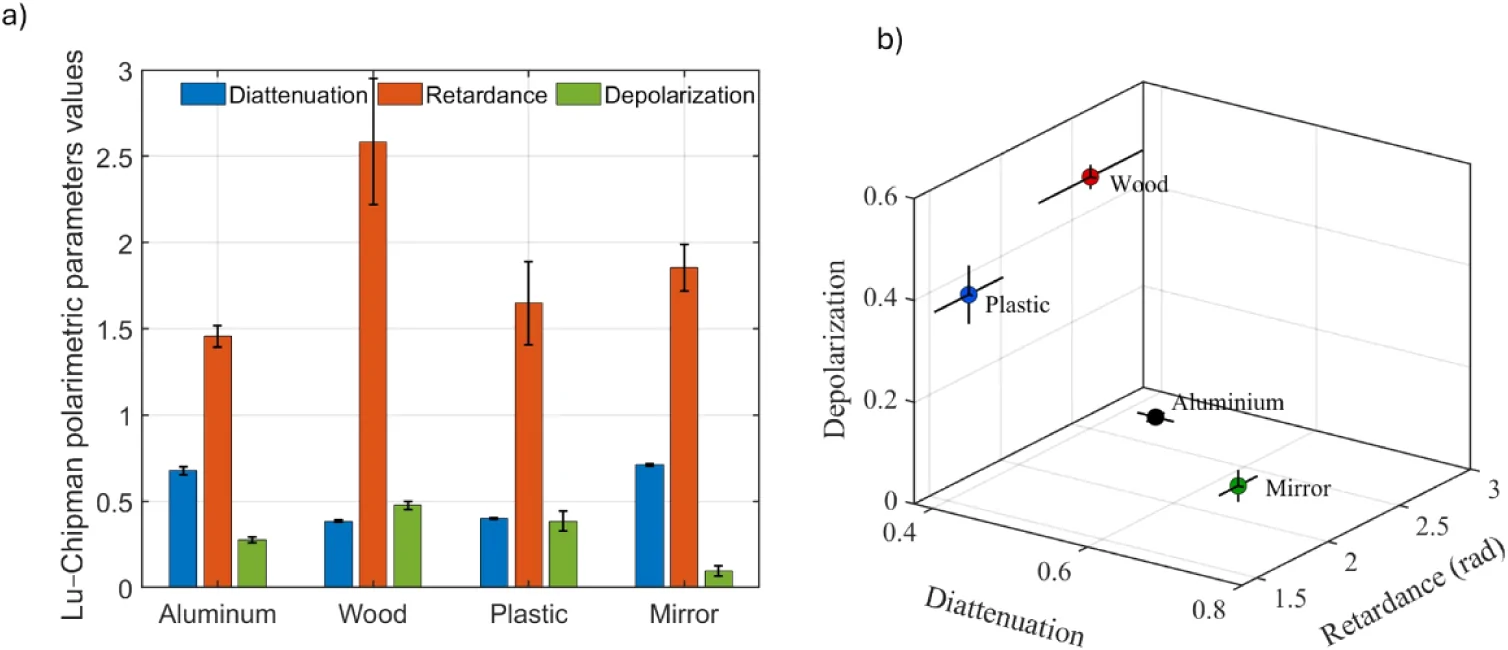
(a) Mean values and standard deviations of diattenuation, retardance, and depolarization extracted using Lu–Chipman decomposition; (b) 3D material fingerprint space extracted using Lu-Chipman decomposition for aluminum, wood, plastic, and mirror. Error bars indicate standard deviation across repeated measurements.

The Lu–Chipman decomposition also provides an internal consistency check for the material interpretation: metallic and mirror-like targets are expected to show stronger diattenuation and lower depolarization, whereas fibrous or volumetric scatterers are expected to show increased retardance or depolarization. The observed trends are consistent with these physical expectations, supporting the interpretation of the dual-comb Mueller measurements as material-specific optical fingerprints.

This decomposition is essential for distinguishing the present approach from empirical polarimetric contrast measurements. Because the dual-comb system retrieves the full Mueller matrix, the measured response can be separated into physically meaningful contributions associated with amplitude anisotropy, phase anisotropy, and depolarizing scattering. The resulting feature space therefore reflects material-dependent light–matter interaction mechanisms rather than instrument-specific intensity variations.

### Discussion and Outlook

The results establish single-cavity dual-comb polarimetry as a practical route to material-aware LiDAR. Beyond providing coherent ranging capability, the polarization-multiplexed dual-comb source acts as an intrinsic, reproducible generator of incident polarization states for full Mueller-matrix recovery. The resulting matrices yield compact material fingerprints that remain consistent across polarimetric purity metrics, Lu–Chipman decomposition, PCA projection, and machine-learning classification. In each representation, specular targets such as mirror and aluminum show stronger polarization retention, whereas wood and plastic exhibit increased depolarization and retardance associated with diffuse scattering, structural anisotropy, or volumetric interaction. This agreement across independent descriptors indicates that the observed separation is governed by material-dependent light–matter interaction rather than by intensity or range contrast alone.

This work should be distinguished from our earlier demonstration of the same single-cavity polarization-multiplexed architecture for ranging, where 340 μm precision was achieved with 10 ms averaging and a 3.8 m ambiguity range.^31^ In that study, distance was extracted from the temporal delay between reference and target beat signals using a 40 MS/s oscilloscope; the submillimeter precision therefore resulted from interferometric delay estimation rather than from the raw sampling interval. The present manuscript instead focuses on polarimetric material identification, showing that the same source architecture can also provide the polarization diversity required for full-vector Mueller-matrix sensing.

The central advantage of the proposed platform is architectural. Conventional active Mueller polarimeters generally require sequential polarization-state generation and analysis using external polarization-state generators and analyzers. Partial-polarization LiDAR systems are simpler, but they usually record only intensity, co- and cross-polarized returns, depolarization ratios, or limited Stokes information, and therefore cannot separate diattenuation, retardance, and depolarization. In contrast, the present dual-comb architecture recovers the complete 4 × 4 Mueller matrix while replacing external polarization switching with the intrinsic polarization beating of the source. These distinctions are summarized in Table 2.

**Table 2:** Advantages of the Dual-Comb Mueller Matrix Polarimetric LiDAR

Approach	Polarization observables	Hardware complexity	Material fingerprint
Partial-polarization LiDAR refs in^1–6^	Intensity, co/cross-polarization returns, linear depolarization ratio, limited Stokes subset	Low (1–2 detectors, fixed analyzer)	Incomplete; cannot separate diattenuation, retardance, and depolarization
Conventional active Mueller polarimetry^1^	Full 4 × 4 Mueller matrix via sequential PSG/PSA states	High (rotating or electro-optic PSG + PSA, synchronization required)	Complete but acquisition speed reduced by sequential switching
**Proposed dual-comb architecture (this work)**	**Full** **4 × 4** **Mueller matrix, intrinsic self-scanning**	**Compact (single-cavity, fast polarimeter only; no PSG or PSA)**	**Complete, physically interpretable, ∼1.185 kHz update rate**

The same source therefore performs several functions that are normally distributed across separate subsystems: illumination, mutual coherence, temporal heterodyne encoding, and polarization-state diversity. Combining these functions in a mechanically static, polarization-multiplexed single-cavity laser reduces alignment sensitivity and simplifies the optical layout. It also ensures that classification is based on interpretable optical descriptors extracted from full Mueller matrices, rather than on black-box intensity features alone. This is particularly important for deployment in environments where material appearance, surface roughness, and illumination geometry can vary substantially.

Several limitations remain. The present measurements were carried out on four representative stationary targets, at a single wavelength, over a short laboratory range, and in a fixed noncoaxial transmitter–receiver geometry. The results should therefore be interpreted as proof-of-concept validation of the self-scanned Mueller feature space rather than as a complete field-ready implementation. Future experiments should include larger and more diverse material libraries, longer stand-off distances, higher optical power, spectrally resolved operation, and outdoor conditions. Imaging or scanning operation will also require stable overlap between the transmitted and received fields across the full angular field of view. This can be achieved either through coaxial integration, using a beam splitter or fiber circulator to transmit and receive through the same aperture, or through a calibrated bistatic geometry with synchronized scan correction for angle and range. Of these options, coaxial integration is the most attractive for scanning LiDAR because it minimizes parallax and simplifies field-of-view alignment.

The key next step is to quantify how incidence angle and detection distance affect Mueller-matrix reconstruction and classification robustness. Changes in viewing geometry modify the effective polarization reference frame, illuminated footprint, Fresnel-weighted s- and p-polarized reflections, and the balance between specular and diffuse scattering. As a result, diattenuation, polarizance, retardance, and depolarization may vary simultaneously, shifting material clusters in the Mueller-derived feature space. Increasing stand-off distance further reduces the collected backscattered power, enlarges the beam footprint, and increases sensitivity to atmospheric attenuation and transmitter–receiver misalignment. Systematic angle- and range-dependent measurements are therefore needed to define the practical operating window of dual-comb Mueller-matrix LiDAR and to determine whether angle-dependent calibration or multiangle training data are required for robust classification. For agricultural and environmental sensing, the most reliable regime is expected to involve moderate viewing angles and short-to-medium stand-off distances, where polarimetric contrast remains high while noise, atmospheric loss, and alignment errors remain manageable.

The architecture is also scalable. Increasing the repetition rate to f_rep_ = 1 GHz and the intercomb offset to Δf = 1 MHz, values demonstrated in microresonator soliton dual-comb platforms^34–36^ and accessible in principle with electro-optic combs or high-repetition-rate mode-locked fiber sources, would reduce the acquisition time to T_acq_ = 1 μs per Mueller matrix. This corresponds to a 1 Mpix/s polarimetric point rate and a coarse unambiguous range of c/2Δ*f* = 150 m. Such a rate is comparable to leading commercial intensity-only 3D LiDAR systems, but with the additional capability of recovering the full 4 × 4 Mueller matrix at each resolved point.^36^ For a 1000 × 1000 point cloud acquired at 1 frame per second, the fast-axis scanner would require a 1 kHz line rate, corresponding to a 500 Hz resonant frequency for bidirectional scanning. This requirement is close to the bandwidth limit of servo-galvanometric scanners but comfortably within the range of MEMS scanners.^37^ These estimates show that the proposed dual-comb Mueller architecture is compatible with state-of-the-art 3D LiDAR acquisition rates while adding full-vector, physically interpretable material contrast.

## Conclusion

In summary, we have demonstrated a self-scanning dual-comb Mueller-matrix LiDAR in which the intrinsic polarization beating of a single-cavity, polarization-multiplexed fiber laser provides the polarization-state basis needed for full Mueller-matrix recovery. This design removes the need for external polarization-state generators and analyzers while preserving access to physically interpretable vectorial information about the target. For four representative materials, the extracted polarimetric-purity and Lu–Chipman descriptors produced clear separation in low-dimensional feature spaces, and PCA-assisted classification achieved 93.75% accuracy. These results establish dual-comb Mueller-matrix sensing as a compact and efficient framework for material discrimination in active polarimetric LiDAR.

## Supplementary Material



## Data Availability

Data underlying the results presented in this paper are not publicly available at this time but may be obtained from the authors upon reasonable request.

## Notes

Dual-comb operating characteristics (Figure S1 (a), (b); representative time-domain Stokes-parameter dynamics (S0–S3) of the dual-comb heterodyne signal measured from (a) aluminum, (b) mirror, (c) plastic, and (d) wood (Figure S2); mean (±standard deviation) of purity indices across five repeated measurements per material (Table S1); PCA variance and feature loadings (Table S2) (PDF)
