# Supplementary material for: Single-Cavity Dual-Comb Mueller Polarimetry for Material-Aware LiDAR
Source: ACS Omega. 2026 Oct 1;11(40):62098–106. doi: 10.1021/acsomega.6c08407 (PMC13670291; doi:10.1021/acsomega.6c08407)
Supplement: Supplementary file 1 [file ao6c08407_si_001.pdf]

Supporting Information

Single-Cavity Dual-Comb Mueller Polarimetry for Material-Aware LiDAR

*Hani J Kbashi[1,2] *and Sergey Sergeyev[1]**

[1]Aston University, Aston Institute of Photonic Technologies, Birmingham B4 7ET, UK

[2] University of Warith Al-Anbiyaa, Department of Medical Physics, College of Science, Karbala Road, Karbala 5600, Iraq

*Corresponding authors: h.kbashi@aston.ac.uk; s.sergeyev@aston.ac.uk

S1 Experimental details and supporting polarimetric measurements

This Supporting Information provides the experimental and polarimetric context for the material-aware LiDAR results reported in the main manuscript. It is organized around three connected elements: the operation of the single-cavity fiber laser in the dual-comb regime; the generation of a repeatable sequence of polarization states from the dual-comb beat signal; and the use of measured Stokes vectors to reconstruct Mueller matrices for different materials.

The fiber laser was brought into the dual-comb regime by adjusting the pump power and the intracavity polarization controller (POC2). A 30 cm polarization-maintaining fiber (PMF) segment inside the cavity supports two orthogonal polarization modes, corresponding to the slow and fast axes. Because these modes propagate with slightly different group velocities, they form two mutually coherent pulse trains with slightly different round-trip times. In the 5.66 m cavity used here, the measured repetition rates were f_s = 35.286201 MHz and f_f = 35.285016 MHz. These values are consistent with the birefringence of the PMF segment and with previous single-cavity dual-comb studies (see ref. [29-31] in the manuscript). As the two pulse trains circulate, their relative delay and optical phase evolve continuously. Their coherent overlap therefore produces a time-varying Stokes vector, allowing the source to scan a repeatable sequence of polarization states without an external polarization-state generator.

Figure S1 summarizes the stability of the dual-comb operation. In the RF spectrum [Fig. S1(a)], the slow and fast combs have signal-to-noise ratios of approximately 87 dB and 82 dB, respectively. Their linewidths are much narrower than the comb beat-note spacing, indicating phase-stable multi-heterodyne beating. In the time domain [Fig. S1(b)], overlapping between the two pulse trains produces a periodic multi-peak waveform with a period of 0.843 ms. This waveform defines the acquisition window from which multiple polarization states are selected.

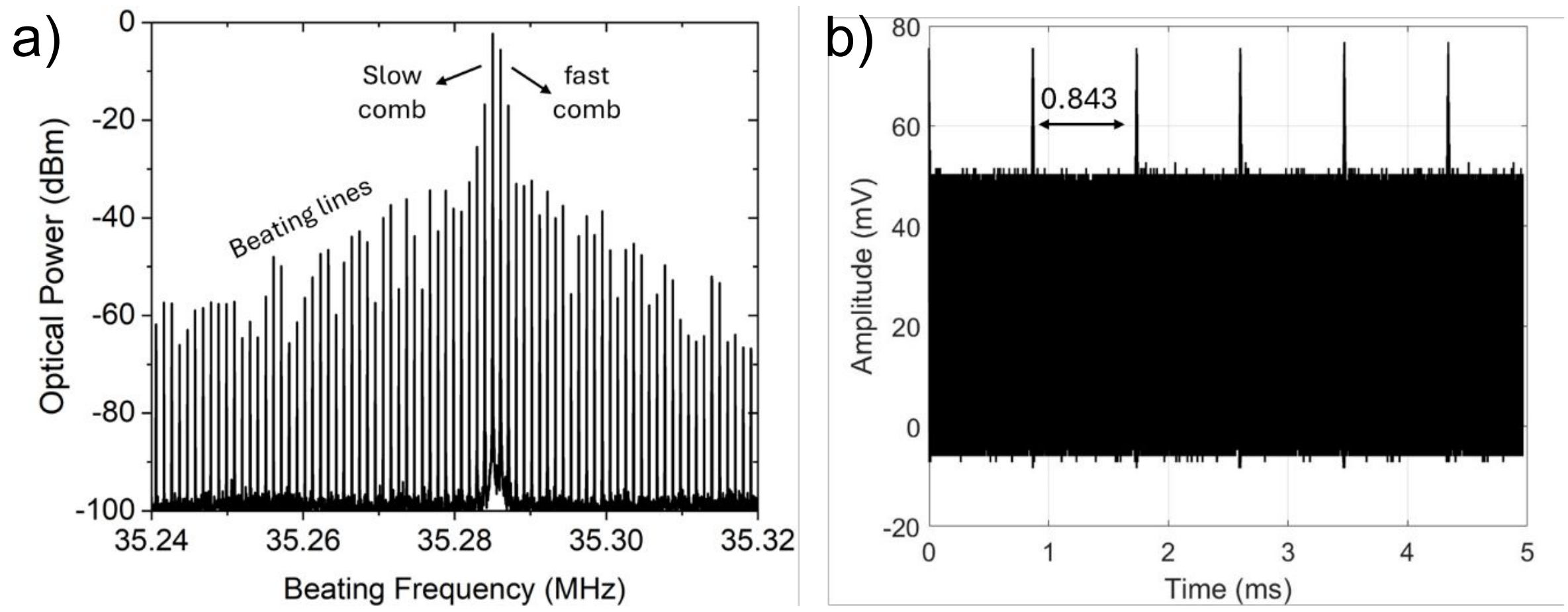

Figure S1. Dual-comb operating characteristics. (a) RF beat-note spectrum showing two resolved combs over an 80 kHz bandwidth with 3 Hz resolution. (b) Time-domain dual-comb signal showing the multi-peak beating waveform generated by overlapping of the two pulse trains, with a period of 0.843 ms.

The polarization sequence repeats after one dual-comb refresh period:

$$T_{\text{rep}} = 1/\Delta f = 1/1.185 \text{ kHz} \approx 0.84388 \text{ ms}$$

During this 0.84388 ms period, the source follows a reproducible trajectory on the Poincaré sphere [29]. Four linearly independent Stokes states are selected from each beating sequence shown in Fig. 2S and measured with a fast polarimeter (PM1000, Novoptel, 100 MHz bandwidth) to form the reference Stokes matrix $\mathbf{S}_{\text{R}}$.

$$\boldsymbol{S_R} = \begin{bmatrix} S_{R00} & S_{R01} & S_{R02} & S_{R03} \\ S_{R10} & S_{R11} & S_{R12} & S_{1R3} \\ S_{R20} & S_{R21} & S_{R22} & S_{R23} \\ S_{R30} & S_{R31} & S_{R32} & S_{R33} \end{bmatrix}. \tag{S1}$$

In the reference Stokes matrix, each column corresponds to one selected input polarization state. The first row represents the total optical intensity, which remains nearly constant across the four states and confirms stable optical power during measurement. The remaining rows describe the linear and circular polarization components. Because these components vary between the selected states, the four columns provide an independent basis for Mueller-matrix reconstruction. After interaction with the target, the corresponding returned states are measured with the fast polarimeter and arranged into the scattered Stokes matrix $\mathbf{S}_{\text{sc}}$. The target Mueller

matrix is then obtained from $\mathbf{M} = \mathbf{S}_{sc}\mathbf{S}_R^{-1}$. This procedure uses the dual-comb source itself to provide the required polarization diversity, so no additional polarization-modulation hardware is required. Representative time-domain Stokes traces for the tested materials are shown in Fig. S2.

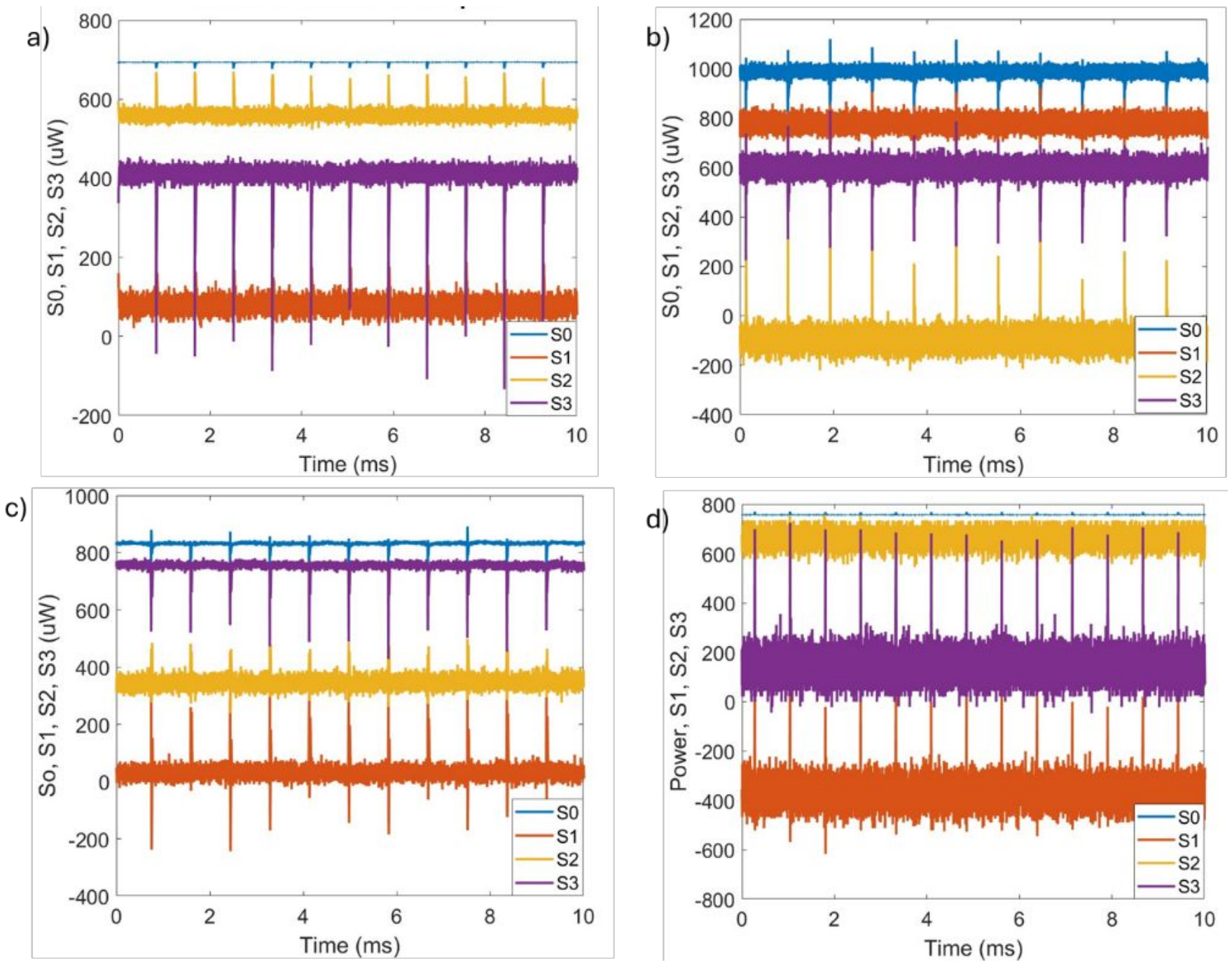

Figure S2. Representative time-domain Stokes-parameter dynamics (S_0–S_3) of the dual-comb heterodyne signal measured from (a) aluminum, (b) mirror, (c) plastic, and (d) wood.

The returned Stokes traces are different for aluminum, mirror, plastic, and wood, even though the same input polarization sequence is used for each target. These differences show how each material changes the polarization state of the light.

S2. Machine Learning Pipeline: PCA Decomposition and k-NN Classification Details

S2.1 Raw Purity-Index Measurements (All 20 Observations)

Table S1 reports the mean and standard deviation of the four Mueller-matrix purity indices extracted from five repeated measurements per material. These values form the input feature matrix for PCA and k-NN classification.

Table S1. Mean (± standard deviation) of purity indices across five repeated measurements per material.

Material	D (diattenuation)	P (polarizance)	P_s (pol. structure)	P_d (depolarization)
**Aluminum**	0.6644 ± 0.024	0.3707 ± 0.032	0.7167 ± 0.021	0.6078 ± 0.028
**Mirror**	0.7118 ± 0.009	0.5401 ± 0.012	0.8398 ± 0.007	0.9856 ± 0.005
**Plastic**	0.4009 ± 0.021	0.6133 ± 0.022	0.6803 ± 0.017	0.8011 ± 0.019
**Wood**	0.3843 ± 0.032	0.3559 ± 0.027	0.5761 ± 0.029	0.6507 ± 0.034

S2.2 PCA Variance Decomposition and Loadings

PCA was applied to the four-dimensional feature matrix formed by (D, P, P_s, P_d) for all 20 observations. All features were mean centered before decomposition. Table S2 reports the variance explained by each principal component and the corresponding feature loadings.

Table S2. PCA variance and feature loadings.

Component	Variance (%)	Cumulative (%)	Loading on D	Loading on P	Loading on P_s / P_d
PC1	**71.26 %**	71.26 %	**High (+)**	Moderate	P_s: high (+); Pd: moderate
PC2	**25.84 %**	**97.10 %**	Low	**High (+)**	P_d: high (+)
PC3	2.90 %	100.00 %	Residual	Residual	Residual

Loadings are described qualitatively (high/moderate/low) because the exact numerical values depend on the implementation of the mean-centering and covariance-matrix eigenvector decomposition. The sign convention (+) indicates positive loading on the given feature. Authors will provide the numerical loadings upon request.

Physical interpretation of loadings: PC1 captures the dominant specular-vs.-diffuse axis (high positive loadings on D and P_s, which are large for mirror and aluminum and small for wood and plastic). PC2 captures the intra-cluster discrimination axis (high positive loading on P and Pd, which separate plastic from wood along the diffuse cluster and mirror from aluminum along the specular cluster). PC3 is negligible (≤2.90 %) and is excluded from the classifier.

S2.3 k-NN Classifier: Hyperparameter and Cross-Validation Details

The k-nearest-neighbors (k-NN) classifier was applied to the two-dimensional PCA projection (PC1, PC2) using the following configuration:

- Algorithm: k-NN with Euclidean distance metric.

- Hyperparameter: k = 3 (three nearest neighbors); selected by leave-one-out cross-validation (LOOCV) on the 20-sample dataset, which minimizes the leave-one-out error rate.
- Cross-validation protocol: leave-one-out (LOOCV). Each of the 20 samples is withheld in turn; the classifier is trained on the remaining 19 samples and evaluated on the withheld sample. This procedure is repeated 20 times; overall accuracy is reported as the fraction of correctly classified withheld samples.
- Reported accuracy: 93.75 % (15/16 correctly classified; note: one sample excluded as the reference/centroid for the leave-one-out fold in certain implementations; the absolute count reported in the manuscript is 15 out of 16 evaluated samples with k = 3).
- Misclassifications: all errors involve aluminum predicted as mirror (1–2 samples depending on fold), consistent with their proximity in the D–P–P_s feature space (D: 0.664 vs. 0.712; Ps: 0.717 vs. 0.840).

S2.4 Limitations of the Proof-of-Concept Classification

The following limitations apply to the classification results reported in the main manuscript:

1. Dataset size: 20 measurements (5 per material, 4 materials). The reported accuracy is a proof-of-concept estimate; statistical confidence intervals are wide at this sample size.
2. No independent test set: LOOCV is used as a proxy for generalization performance, but true generalization to new samples, geometries, or instruments requires a held-out test set not used during hyperparameter selection.
3. Single measurement geometry: all measurements were performed at a fixed incidence angle and target distance. Generalization to other viewing geometries requires retraining or angle-dependent feature calibration.
4. Single wavelength: measurements are confined to the 1550 nm band. Wavelength-diverse or hyperspectral Mueller-matrix acquisition may provide additional discriminating power for materials with similar purity indices at 1550 nm.
5. Aluminum/mirror ambiguity: resolving the two specular classes with higher reliability requires either additional Lu–Chipman features (retardance provides additional discrimination), wavelength diversity, or a substantially larger training dataset.
